# The Importance of Community Voice: Using Community-Based Participatory Research to Understand the Experiences of African American, Native American, and Latinx People During a Pandemic

**DOI:** 10.5888/pcd20.220152

**Published:** 2023-03-09

**Authors:** Amanda Haboush-Deloye, Erika Marquez, Rebecca Dunne, Jennifer R. Pharr, for the Nevada Minority Health and Equity Coalition

**Affiliations:** 1 Nevada Institute for Children’s Research and Policy, School of Public Health, University of Nevada, Las Vegas; 2 Department of Environmental and Occupational Health, School of Public Health, University of Nevada, Las Vegas

## Abstract

**Introduction:**

Although the disproportionate impact of COVID-19 infection, hospitalization, and death rates on racial and ethnic minority communities in the US is known, information about how COVID-19 has affected these communities and how community context and perceptions can inform a better response to future health crises needs further exploration. To help achieve these objectives, we used a community-based participatory research approach to gain a better insight into African American, Native American, and Latinx communities.

**Methods:**

From September through December 2020, we conducted 19 focus groups and recruited 142 participants. Participants were selected via a purposeful sampling technique. We used a phenomenology study design to conduct semistructured interviews, thematic analysis to code qualitative data, and descriptive statistics to summarize demographic data.

**Results:**

Data analysis revealed the following 3 themes: 1) COVID-19 exacerbated mistrust, anxiety, and fear in racial and ethnic minority populations, affecting their mental health, 2) understanding sociocultural context is essential for emergency response, and 3) adapting communication strategies can help address community concerns.

**Conclusion:**

Amplifying the voices of people disproportionately affected by the COVID-19 pandemic can help to inform a better response to future health crises and ultimately reduce health inequity among racial and ethnic minority populations.

**Summary**

**What is already known on this topic?**

COVID-19 has exacerbated long-standing health disparities in racial and ethnic minority populations. We used community-based participatory research to examine the experiences of African American, Native American, and Latinx people to improve responses to public health emergencies.

**What is added by this report?**

The following themes emerged: public health crises have detrimental impacts on mental health because of historical and current traumas; sociocultural context must be understood to mitigate disparities; and communication strategies reflecting sociocultural context can help address community concerns.

**What are the implications for public health practice?**

Public health strategies are at the forefront of protecting community health. Study findings provide approaches and considerations to inform how we engage and mitigate risk in communities that are vulnerable to health disparities.

## Introduction

In the US, hospitalizations due to COVID-19 have been 2.6 times higher among African American people, 2.5 times higher in Latinx people, and 3.3 times higher in Native American people than among non-Hispanic White people; additionally, fatalities due to COVID-19 have been higher in these populations (1). Reduced access to quality health care, inflexible low-wage employment, lack of paid sick leave, overrepresentation in correctional and immigration detention facilities, and overcrowded low-quality housing (2) are a few of the long-standing structural inequities that have contributed to higher rates of COVID-19 infection, hospitalization, and death in racial and ethnic minority populations in the US (3–5).

To reduce health disparities exacerbated by COVID-19’s disproportionate impact on racial and ethnic minority communities, it is imperative to first understand their experiences and needs. One way to engage diverse communities is by supporting and amplifying their voices through community-based participatory research (CBPR) (6). CBPR strives to prioritize equity in the relationship between researchers and community-based organizations by practicing principles of co-learning, ensuring efforts have a mutual benefit, and making a long-term commitment (7). CBPR has been used across disciplines and issues to improve community outcomes (8). It provides the foundational principles to understand community context and concerns, informs strategies to better prepare for emergencies, and communicates risk among communities that are underresourced in terms of such necessities as food, clothing, shelter, and access to health care during times of crisis (9).

The objectives of this study were to describe how 3 racial and ethnic minority populations in Nevada were affected by COVID-19 and how community context and perceptions can inform a better response to future health crises.

## Methods

This study was part of a larger project initiated by the Nevada Minority Health and Equity Coalition (NMHEC), a partnership of academic, civic, private, and community organizations that dedicated efforts to conduct education and outreach in key communities in Nevada affected by COVID-19. We used a CBPR approach to conduct focus groups with members of populations hit hard by the pandemic — African American, Native American, and Latinx people (1). We examined the impact of COVID-19 in these populations, perceptions of risk-mitigation measures such as contact tracing, and perceptions of the COVID-19 vaccine. The study was reviewed and approved by the institutional review board at the University of Nevada, Las Vegas.

### Study setting

Since the late 1990s, Nevada has been the fastest-growing state; it had a population of more than 3.1 million in 2021. Nevada has 17 counties; 3 are urban, 3 are rural, and 11 are frontier (10). Frontier counties are more remote than rural counties in terms of travel time and distance from the nearest population center with specialized medical care facilities. Approximately 74.3% of the population resides in Clark County (Las Vegas metropolitan area) (10). Nevada is the third most racially and ethnically diverse state in the country (11). Even before COVID-19, disparities existed in racial and ethnic populations: higher rates of poverty, lower rates of high school graduation, less access to health care and nutritious foods, and higher rates of diabetes, heart disease, and infant mortality (12). These disparities demonstrate the negative impact of long-standing inequities in the social determinants of health, which increase the risk for communicable diseases such as COVID-19. Populations most vulnerable to COVID-19 should be prioritized for receiving accurate information in an emergency response.

Initial communication about COVID-19 in Nevada was headed by federal and state government officials and relied heavily on social media and television news outlets. However, many people in Nevada do not have access to a computer at home or the internet, and their main source of communication is a smartphone. Race and income are the strongest predictors of the presence of the internet, indicating that populations most vulnerable to illness may not receive vital information (13).

We used a CBPR approach to frame our principles. We contracted with community-based organizations rooted in and trusted by the populations they serve. These organizations provide such services as advocacy, peer support, and distribution of resources (eg, food, clothing, shelter). Partner organizations participated in discussions to develop focus group questions, recruit community members, co-facilitate focus groups, and assist with data analysis and dissemination. Information gathered from focus groups informed the development of culturally responsive and linguistically appropriate material in multiple languages. 

### Study design

We selected a phenomenology study design, the goal of which is to describe the lived experience of a phenomenon of interest, to clarify the meanings participants gave to the impact of COVID-19, risk-mitigation measures, and perceptions of the COVID-19 vaccine. We conducted semistructured focus groups to examine the experiences of participants and capture commonalities and differences among the 3 focal populations (14).

### Participant recruitment

Beginning in September 2020, we used purposeful sampling to recruit participants (15). Community-based organizations, each funded for its work, helped to recruit focus group participants. Focus groups were advertised through social media, flyers at locations still operating in-person, and personal outreach. All advertisements stated the following inclusion criteria: residence in Nevada, identification with their respective racial or ethnic group, and aged 18 years or older. There were no additional inclusion or exclusion criteria. A $20 gift card was offered for participation.

### Data collection and analysis

We conducted 19 focus groups from October through December 2020. Most focus groups were held via Zoom, but because of limited digital access, 3 focus groups with Latinx participants were held in person at a community-based organization. Research staff members followed all COVID-19 safety policies in place at the time. During each session, facilitators included at least 1 individual from the community-based organization who had received training on facilitation of a focus group and 2 research staff members from the Nevada Institute for Children’s Research and Policy. One staff member co-facilitated and the other took notes and provided support. At the beginning of each session, a short summary of the purpose of the focus group was stated, “community” was defined as referring to the race or ethnicity with which the participants identified, permission to record was asked, and a reminder that their name would not be connected to any of their responses was given. Focus group sizes ranged from 3 to 14 participants. Typically, a focus group lasted for 1 hour and 30 minutes.

All participants were asked to complete a brief questionnaire that asked about age, race and ethnicity, level of education, sex/gender, whether the participant rents or owns their home, and annual household income. Although the questionnaire included transgender and gender fluid/nonbinary as choices, no one selected these categories. We did not collect identifying information on this form, and noncompletion did not exclude a participant from receiving a gift card. The semistructured interview included questions (Box) on the participants’ personal experiences with COVID-19 and opinions on testing, contact tracing, and a potential vaccine.

**Box. Semistructured Focus Group Questions Designed to Elicit Information on COVID-19 Experiences Among Participants From 3 Priority Populations — African American, Native American, and Latinx — in Las Vegas, Nevada, October–December 2020**

**Topics and questions**

Topic 1: How has COVID-19 impacted community members?

- If you are comfortable sharing, we would like to know how COVID-19 has impacted you and your community.
- What are some challenges that you may have faced?
- How do you think that you and your community might have been impacted differently compared with others because of your race/language barrier/immigration status?
- How do you think members of your community might be more at risk for COVID-19?

Topic 2: Case investigation and contact tracing

- What are your perceptions about case investigation and contact tracing? What are some concerns?
- There are some options for testing and tracking people who may have COVID-19 to help slow the spread of the virus. We are going to review a few of the options. For each, can you talk about if you would use it? And what concerns you have about it?
- What information do you think we could share with members of the community to help address the negative perceptions about case investigation and contact tracing?
- Overall, what cultural beliefs in your community may prevent someone from getting tested for COVID-19 or from participating in contact tracing?

Topic 3: Opinions and planned behaviors regarding COVID-19 vaccine or vaccine hesitancy

- Currently, companies are working on a COVID-19 vaccine. How do you feel about a potential vaccine?
- What cultural beliefs in your community may prevent someone from getting the vaccine?
- What trusted sources of information do you use to learn more about health care, COVID-19, treatments, and vaccines?
- What factors or messages are important in talking to members of the priority population about a vaccine?
- Is there anything else you would like to share about your community about COVID-19, testing, contact tracing, or a potential vaccine?

We used SPSS version 28 (IBM Corporation) to generate descriptive statistics on demographic data. After research staff members transcribed the recorded focus groups and the sessions conducted in Spanish were translated into English, we used thematic analysis to analyze the qualitative data. Ongoing, inductive line-by-line coding was performed independently by 2 members of the research team to ensure intercoder reliability. Group meetings with the entire research team were held to discuss coding discrepancies, and consensus was achieved when at least 3 of the 4 staff members agreed. To make the data accessible to our community partners, all coding and analysis was conducted in Excel (Microsoft Corporation). Once codes were finalized, the research team worked together to identify commonalities, differences, and relationships among participant responses for each question and produce a summary of themes for each population (16). Next, the information was provided to the community partner organizations for review and feedback. Suggested changes were discussed and agreed upon with the community partner and the research team. The summaries were then sent to the focus group participants for feedback.

## Results

A total of 142 individuals (35 African American, 29 Native American, and 78 Latinx) participated in 1 of 19 focus groups; 98 (69.0%) participants completed the questionnaire (Table 1). 

**Table 1:** Demographic Characteristics of Participants in Focus Groups Designed to Elicit Information on COVID-19 Experiences Among Participants From 3 Priority Populations — African American, Native American, and Latinx — in Las Vegas, Nevada, October–December 2020^a^

Variable	Latinx (n = 78)	African American (n = 35)	Native American (n = 29)
Submitted demographic form	63	23	12
**Age, y**			
18–30	8 (12.7)	7 (30.4)	6 (50.0)
31–50	38 (60.3)	9 (39.1)	1 (8.3)
51–64	12 (19.0)	3 (13.0)	4 (33.3)
≥65	2 (3.2)	2 (8.7)	0
Prefer not to answer	3 (4.8)	2 (8.7)	1 (8.3)
**Education level**			
Less than 9th grade	10 (15.9)	0	0
9th to 12th grade, no diploma	8 (12.7)	0	0
High school diploma or GED	18 (28.6)	1 (4.3)	4 (33.3)
Some college, no degree	9 (14.3)	5 (21.7)	4 (33.3)
College degree	14 (22.2)	17 (73.9)	4 (33.3)
Prefer not to answer	4 (6.3)	0	0
**Sex/gender**			
Male	1 (1.6)	7 (30.4)	3 (25.0)
Female	61 (96.8)	16 (69.6)	9 (75.0)
Prefer not to answer	1 (1.6)	0	0
**Income, $**			
0	3 (4.8)	1 (4.3)	0
<14,999	10 (15.9)	1 (4.3)	0
15,000–24,999	8 (12.7)	0	2 (16.7)
25,000–39,999	11 (17.5)	2 (8.7)	2 (16.7)
40,000–54,999	4 (6.3)	6 (26.1)	1 (8.3)
55,000–79,999	1 (1.6)	4 (17.4)	2 (16.7)
80,000–109,999	2 (3.2)	5 (21.7)	1 (8.3)
≥110,000	1 (1.6)	2 (8.7)	2 (16.7)
Prefer not to answer	23 (36.5)	2 (8.7)	2 (16.7)
**Home**			
Rent	42 (66.7)	15 (65.2)	5 (41.7)
Own	12 (19.0)	8 (34.8)	5 (41.7)
Prefer not to answer	9 (14.3)	0	2 (16.7)

Abbreviation: GED, General Educational Development.

a All values are number (percentage) unless otherwise indicated.

The data analysis revealed 3 key themes: 1) COVID-19 exacerbated mistrust, anxiety, and fear in racial and ethnic minority communities, affecting their mental health, 2) understanding sociocultural context is essential for emergency response, and 3) adapting communication strategies can help address community concerns (Table 2). 

**Table 2:** Identified Themes and Subthemes in Focus Groups Designed to Elicit Information on COVID-19 Experiences Among Participants From 3 Priority Populations — African American, Native American, and Latinx — in Las Vegas, Nevada, October–December 2020

Theme and subtheme	Latinx	African American	Native American
**COVID-19 exacerbated mistrust, anxiety, and fear among racial and ethnic minority populations, affecting their mental health**			
Increases in the following: fear, anxiety, stress, and social isolation	■	■	■
Fear of getting sick and leaving the house	■	■	■
Cancellation of activities pivotal to well-being	■	■	■
Increases in violence	■		■
Increases in protests and blatant displays of racism		■	
Concerns of discrimination	■	■	■
Historical trauma		■	■
Lack of trust	■	■	■
**Understanding sociocultural context is essential in an emergency response**			
Economic challenges	■	■	■
Immigration status	■		
Crowded home conditions	■		■
Concept of “house”			■
Superstition		■	
Machismo	■		
Traditional medicine	■	■	■
Spiritual or religious beliefs	■	■	■
**Adapting communication strategies can help address community concerns**			
Do not lie to the community; provide both the pros and cons of the vaccine	■	■	■
What are the side effects? Will the vaccine make someone sick?	■	■	■
What are the ingredients of the vaccine?	■		
Be transparent; want to see the science behind it		■	
Don't allow anybody to intimidate or force them to do anything; choice		■	
If you get the vaccine, you protect yourself and the ones you love	■		■
Material needs to be in the native language	■		

### Theme 1: COVID-19 exacerbated mistrust, anxiety, and fear in communities of color, affecting their mental health

African American, Native American, and Latinx participants reported increases of fear, anxiety, stress, and social isolation in light of the COVID-19 pandemic. “The truth is that this disease brings fear, it brings anguish, it brings anxiety, it brings hunger, it brings a lot of hunger, a lot of despair, depression, anxiety, so this are difficult times” [Latinx 1].

Among Latinx and African American participants, these feelings were attributed to the rapid shutdown and lack of supplies in stores at the beginning of the pandemic. Fear and anxiety about getting sick personally, having a friend or family member become sick, and leaving the house, were common across all groups. In all groups, participants discussed how their fear and anxiety of becoming ill and leaving the house resulted in prolonged home confinement. Participants struggled with the cancelations of activities that were pivotal to their well-being, such as church services, celebrations of achievements, and the practice of cultural traditions, such as the sweat ceremony for Native Americans. In addition, Native American participants noted that social isolation had been especially taxing on the mental health of elders, who might live alone in remote areas. Participants in all groups indicated that this social isolation for long periods of time negatively affected their health, even to the point of hospitalization for 1 individual.

Latinx and Native American participants reported additional stressors, such as concern about increases in violence in the community and the home. These conversations highlighted the need for additional treatment services for mental health and substance use. African American participants noted that protests and blatant displays of racism in their communities contributed to overall stress levels. Similarly, Latinx participants voiced concerns about discrimination as a stressor. For example, 1 participant noted that coworkers maintained their distance and made the participant feel rejected because of having contracted COVID-19. Another participant reported that after having an asthma coughing episode, *“*This cashier sprayed Lysol on my face and told me, ‘You have coronavirus’” [Latinx 2].

Historical trauma, mistreatment, and discrimination continue to fuel feelings of distrust, which affects COVID tracing, testing, and vaccination. For example, participants did not want to get tested or share test results, because they were worried about being treated poorly — “like a leper” — if they had a positive test result. African American and Native American participants reiterated the lack of trust in the health care system and historical trauma as contributing to these concerns. One participant noted, “I cannot think of anything that you could do to get people to trust the government as far as when it comes to health” [African American 1].

Another participant stated the following:

So, I think they have a really hard sell within our community specifically, especially with the elders, the people that really need it the most. They’ve witnessed things firsthand that the government has done to it. So, it’s going to be really hard to convince them, hey, you can trust them this time [Native American 1].

These feelings of distrust were applied to risk-mitigation measures by all participants. In discussions about contact tracing apps, participants were concerned that reports of COVID-19 cases in their location would not be accurate because they relied on self-report. An additional fear was that the app could be hacked and personal information stolen, resulting in an invasion of privacy.

Participants had qualms about vaccination. All participants voiced not wanting to be experimented on or the first to receive a vaccine, to avoid treatment as “guinea pigs” [African American 2, 3, 4, 5, 6, 7, 8]. The historical trauma from the Tuskegee experiments [African American 2, 9, 10, 11, 12, 13] and the spread of smallpox by gifted blankets [Native American 2] contributed to fear of the vaccine. In addition, Latinx and African American focus group participants worried that a government chip with tracking capabilities could be introduced into their body through the COVID-19 vaccine. For many, concerns about testing, tracing, and vaccination contributed to feelings of stress and anxiety because they perceived no relief from the pandemic.

### Theme 2: Understanding sociocultural context is essential in an emergency response

Economic challenges were reported across all groups. A common concern was the difficulty in obtaining unemployment benefits; even if obtained, these benefits would often fail to cover all living expenses. A loss of employment or a reduction in hours would also result in a loss of health insurance coverage, which unemployment benefits do not provide. Immigrant Latinx participants highlighted the challenges of citizenship status and ineligibility for benefits such as unemployment benefits. These communities relied on well-organized agencies to provide basic needs. Many living in multigenerational homes expressed concerns about keeping their family members safe, especially when risk-mitigation measures were not practical. Safety was also a concern for those living in overcrowded housing conditions, a marker of the financial crisis (African American and Native American). Native American participants expressed an increased concern about exposure to COVID-19 because their concept of *house* expands beyond the physical location where one lives and includes the spaces of many family members.

Additional cultural factors that affected use of risk-mitigation measures included superstitions, behavioral expectations, traditional medicine, and spiritual or religious beliefs. For example, some African American participants noted that talking about an illness will get you sick, resulting in people not talking about COVID-19. Machismo was reported among Latinx participants as a cultural belief that might discourage men from getting vaccinated for fear of being viewed as weak. Relying on traditional medicine as protection from or as a remedy to cure COVID-19, rather than modern medicine (eg, COVID-19 vaccine), was mentioned among all participants as a cultural belief that could potentially hinder risk-mitigation strategies. “I come from a traditional family who believes in the herbs and the medicines that we have” [Native American 2].

Resistance to risk-mitigation measures was further discussed in terms of spiritual and religious beliefs. “God got me and I’m not gonna do it” [African American 5]. “The creator will protect us” [Native American 3]. “What we need is having a lot of faith in God” [Latinx 3].

Focus group sessions among all participants revealed the existence of cultural beliefs influencing perceptions of contact tracing, testing, and vaccination, further highlighting the importance of understanding sociocultural context in an emergency response.

### Theme 3: Adapting communication strategies can help address community concerns

One focus group question asked about messages that were important for communicating with members of the priority population about a vaccine. All focus group participants expressed the notion of reassuring community members that the vaccine was “tried, tested, and true,” safe to use, and backed by science. African American, Latinx, and Native American participants discussed the importance of providing a comprehensive overview of what to expect when obtaining the vaccine (eg, side effects, duration of side effects). African American participants stated that messaging should not force anyone to get the vaccine and that doctors need “to hear us out” — and communicate risks before benefits when discussing the vaccine with patients. Native American participants indicated that messaging should frame the vaccine as a personal choice and include a preventive approach to COVID-19 that emphasizes using traditional and Western (eg, the vaccine) medicine in tandem. Native American participants discussed multiple media forms for messaging, consisting of recognizable cultural items such as the medicine wheel, buffalo, and wolves. Latinx participants suggested framing messaging related to vaccine uptake in light of community — “if you take care of yourself, you take care of those you love, too” — and indicated that professionals who speak Spanish should help the community effectively communicate information about the vaccine to ensure that messages are clear.

For the focus group question asking community members about the trusted sources of information they use to learn more about health care, African American and Native American focus group participants emphasized the importance of vaccine information “coming from us” — people in racial and ethnic minority groups or respected community members. “There has not been any outreach through Black-led organizations around contact tracing to my knowledge. And that is very disconcerting to me . . . so I am afraid that at least in the African American community that we have kind of been left out of the puzzle” [African American 14].

Latinx participants discussed their trust in local institutions, such as the Mexican Consulate in Las Vegas, a COVID-19 health campaign called Esta En Tus Manos Nevada, and the news station Primera Hora. All focus group participants cited people in health care or the medical field (eg, their primary care doctor, family pediatrician, specialists, health care professionals) as trusted sources of information. Although mistrust of the government due to historical events was discussed by African American and Native American participants, some African American participants cited the Centers for Disease Control and Prevention (CDC) as a trusted source. Latinx participants also cited establishments such as the local health department, the World Health Organization, and CDC as trustworthy sources for vaccine information. Native American focus group participants talked about having trust in health officials but not the government. All focus group participants indicated they would trust the experiences of close family members and friends.

## Discussion

Historically, racial and ethnic minority populations are confronted with factors and events that systematically and disproportionately affect the well-being of their families and communities. Although public health is at the forefront of improving conditions for communities that are vulnerable to health disparities and underresourced in terms of necessities like food, clothing, shelter, and access to health care, more work needs to be done to understand how to better implement strategies to improve community health.

The first aim of our study was to understand the effects of COVID-19 by examining the lived experiences of populations most affected by the pandemic. COVID-19 contributed to uncertainty, worry, concern, and stress across all communities in the US; however, chronic stress is known to place a disproportionate burden on racial and ethnic minority populations (17). Chronic stressors combined with COVID-19 escalated feelings of fear, anxiety, and social isolation in our study population. Participants indicated that racism and discrimination worsened their degree of stress. Some acknowledged increased violence in the home and advocated for more social services. Strategies for alleviating stress and increasing access to mental health services should be incorporated into emergency responses.

The second aim of our study was to understand how social and cultural context could help inform risk-mitigation strategies in racial and ethnic minority populations. One major lesson learned was that context matters. For example, socioeconomic challenges can hinder how risk-mitigation strategies are implemented at home and in the workplace. Individuals in communities vulnerable to health disparities must make tough decisions to meet basic needs, often requiring them to risk exposure to COVID-19 and making it almost impossible to adhere to recommended safety protocols.

The histories and present-day experiences of collective trauma among racial and minority populations continue to validate a mistrust in systems; mistrust in the health care system plays a role in emergency response strategies. Distrust of the health care system, health care providers, and treatments is not a novel concept, and it is more prevalent among racial and ethnic minority populations than among White populations in the US (18). Furthermore, lack of trust contributes to racial and ethnic health and health care disparities, resulting in a decreased likelihood of engaging in various health behaviors (18). Our findings support the concept that discrimination influences medical mistrust, thereby worsening the impact of COVID-19 on racial and ethnic minority populations (19). Inadvertently, this distrust spills over into the implementation of risk-mitigation measures, including vaccination. Understanding sociocultural context is important to understand how or why communities do or do not adopt certain health behaviors.

The CBPR model is adept at addressing community issues along with a community during an emergency response and incorporating sociocultural context to enhance communication and engagement. We used this approach to prioritize community concerns across our 3 priority populations and reached consensus on how to develop COVID-19 information communication strategies to reduce concern and address misinformation, while working with community members directly affected. This work led to the implementation of diverse communication strategies to support COVID-19 risk mitigation and communications. Public health response to future pandemics should use diverse strategies. Emergency responders can start by building trust and committing to customizing educational materials to the needs of communities.

Our findings indicate the critical need to build trust. We need to make efforts to engage and maintain involvement from once-exploited communities by establishing bidirectional trust and participation strategies that fully demonstrate respect and inclusion (20). Broadly speaking, public health professionals should begin forming partnerships with people and organizations in racial and ethnic minority communities now and involve them in current planning and decision-making in a consistent manner, not only in times of a national or worldwide crisis. A relationship model, in which public health decision makers share power with communities so that communities have an opportunity to voice what they need and when they need it, is a critical component of building and maintaining respect. Additionally, because of this existing, continued, and trusted relationship, the community can immediately partner with public health professionals when making decisions during an emergency response.

Second, a one-size-fits-all strategy to emergency response communication is not effective at reaching diverse communities that often lack necessities and access to health care. Our 3 priority populations had similar concerns about physical and historical barriers to vaccine uptake. Community partners should be involved in developing methods of communication and design of materials and have input in the selection of trusted messengers. Historical missteps and the degree to which previous forms of engagement between communities and public health professionals has worked within the community must be acknowledged. In health-related matters, cultural competency — having knowledge of a group’s cultural perspectives and typical behaviors or beliefs — is necessary to bridge cultural boundaries (20). For example, communication with racial and ethnic minority communities should be in their preferred language (21), culturally relevant, and contain culturally appropriate colors, images, and pictures (22).

In addition, risk-mitigation and treatment strategies should be designed and communicated in a way that integrates Western approaches with racial or ethnic cultural health practices. For example, the use of sage (or other traditional remedies) for cleansing in tandem with encouraging vaccination might better honor the healing practices of Native Americans.

By incorporating these cultural considerations, gaps in mitigation messaging are less likely to occur, risks can be responded to more efficiently, and prevention can be more streamlined (23). Furthermore, taking a meaning-centered approach to understanding community members can reveal how external social, economic, and political forces affect the community (20). Including members of these populations as partners to create appropriate communication for their people will help ensure that their voice and values are reflected in the content and will likely be better received by the community. In paying attention to how language is used and showing respect for culture, community-engaged approaches are likely to operate more smoothly.

### Limitations

This study has several limitations. Because we conducted most focus groups virtually, participation may have been limited among populations with lack of access to or little knowledge of using technology. Therefore, our results may not be generalizable to other groups facing barriers to accessing technology. Next, given that all participation was based on the willingness of individuals to participate, there could be something different about those who participated compared with those who chose not to participate. Lastly, although men from the 3 priority populations participated, most participants were women, limiting generalizability.

### Conclusion

The use of CBPR principles assisted the research team and community-based organizations in gathering valuable information on how COVID-19 affected 3 racial and ethnic minority groups and some immediate and long-term needs to reduce disparities. Because the voices of African American, Native American, and Latinx populations are often excluded from the decision-making process, engaging in this type of qualitative research is essential for amplifying their voices, improving population health outcomes, and curbing health inequities. CBPR provides a platform to share power in all aspects of an emergency health response by providing a “rightful” seat at the table to discuss and drive implementation measures to address community concerns. An increase in policies requiring the use of CBPR in emergency response as well as other critical public health endeavors (eg, needs assessments, strategic planning) could lead to increased community integration and an elimination of health disparities.
